# Extraction Techniques for Polycyclic Aromatic Hydrocarbons in Soils

**DOI:** 10.1155/2010/398381

**Published:** 2010-04-12

**Authors:** E. V. Lau, S. Gan, H. K. Ng

**Affiliations:** Faculty of Engineering, The University of Nottingham Malaysia Campus, Jalan Broga, Semenyih, Selangor Darul Ehsan 43500, Malaysia

## Abstract

This paper aims to provide a review of the analytical extraction techniques for polycyclic aromatic hydrocarbons (PAHs) in soils. The extraction technologies described here include Soxhlet extraction, ultrasonic and mechanical agitation, accelerated solvent extraction, supercritical and subcritical fluid extraction, microwave-assisted extraction, solid phase extraction and microextraction, thermal desorption and flash pyrolysis, as well as fluidised-bed extraction. The influencing factors in the extraction of PAHs from soil such as temperature, type of solvent, soil moisture, and other soil characteristics are also discussed. The paper concludes with a review of the models used to describe the kinetics of PAH desorption from soils during solvent extraction.

## 1. Introduction

Polycyclic aromatic hydrocarbons or polynuclear aromatic hydrocarbons (PAHs) are compounds produced through incomplete combustion and pyrolysis of organic matter. Both natural and anthropogenic sources such as forest fires, volcanic eruptions, vehicular emissions, residential wood burning, petroleum catalytic cracking, and industrial combustion of fossil fuels contribute to the release of PAHs to the environment [1]. The presence of PAH compounds in soils is an issue of concern due to their carcinogenic, mutagenic, and teratogenic properties. In 2008, 28 PAHs have been identified as priority pollutants by the National Waste Minimization Programme, a project which is funded by US Environment Protection Agency [2].

 PAHs which consist of fused benzene rings are hydrophobic in nature with very low water solubility and high octanol-water partition coefficient (*K*_ow_). Hence, they tend to adsorb tightly to organic matter in soil rendering them less susceptible to biological and chemical degradation. Prolonged aging time in contaminated soil promotes the sequestration of PAH molecules into micropores and increases the recalcitrance of PAHs towards treatment [3]. Thus the extraction process of PAHs from soil for analysis is made more complicated due to these factors. In this paper, various analytical extraction techniques for PAHs in soils will be reviewed, ranging from more widely applied methods such as Soxhlet extraction, sonication, mechanical agitation, and accelerated solvent extraction to alternative ones such as supercritical and subcritical fluid extraction, microwave-assisted extraction, solid phase extraction and microextraction, thermal desorption and flash pyrolysis, as well as fluidised-bed extraction. The influencing factors in the extraction of PAHs from soil such as temperature, type of solvent, soil moisture and other soil characteristics are also discussed. Finally, a review of the models used to describe the kinetics of PAH desorption from soils during solvent extraction will be provided.

## 2. Extraction Techniques

### 2.1. Soxhlet Extraction

The Soxhlet extraction has been vastly used as a benchmark technique in the extraction of PAHs from soils and sediments. Basically, in the Soxhlet extraction technique, the solid sample is placed into an extraction thimble which is then extracted using an appropriate solvent via the reflux cycle. Once the solvent is boiled, the vapour passes through a bypass arm into the condenser, where it condenses and drips back onto the solvent in the thimble. As the solvent reaches the top of the siphon arm, the solvent and extract are siphoned back onto the lower flask whereby the solvent reboils, and the cycle is repeated until all the sample is completely extracted into the lower flask.

The main disadvantage of this extraction process is the use of large volumes of solvent, which can be more than 150 mL for the extraction of PAHs from a mere 10 g of soil sample. In addition to that, this method is very labour intensive and time consuming, as the solvent has to be refluxed up to 24 hours to achieve considerable extraction efficiencies [4, 5]. The Soxhlet extraction too has been shown to have relatively poor selectivity for PAHs compared to bulk soil organic matter, with approximately a quarter to one third of bulk soil organic matter removed during extraction [6]. Studies have indicated that the chromatograms of extracts produced via Soxhlet using GC-MS and GC-FID yielded more artefact peaks with branched alkane “humps,” demonstrating that compounds such as n-alkanes and humic substances other than PAHs are coextracted using the Soxhlet technique [6, 7]. Other minor drawbacks of using the Soxhlet apparatus include the likelihood of sample carryover, the need to fractionise extracts to avoid heavy contamination of GC injection port, and the unfeasibility of redissolving dried Soxhlet extracts [8, 9]. 

Nonetheless, the Soxhlet extraction is still the preferred method because of its comparative extraction results despite the nature of matrix sample. Not only does the Soxhlet extraction yields similar results with methods such as the supercritical fluid extraction (SFE), microwave-assisted extraction (MAE), accelerated solvent extraction (ASE), and ultrasonic methods, but the results also show small variations with low relative standard deviations [10–12]. Statistically, Berset et al. [12] showed that the Soxhlet method resulted in median values which corresponded to the overall mean of other extraction procedures including ASE, SFE, MAE and sonication. The efficiency of the Soxhlet extraction increases with molecular weight, reaching an efficiency range of 84–100% for PAHs with more than 4 rings [13].

 To further improve the Soxhlet extraction technique, Edward Randall patented the automated Soxhlet extraction method in 1974. This is a two-step procedure which combines boiling and rinsing such that the total extraction time is reduced while the evaporated solvent condenses rapidly for reuse, reducing the amount of total solvent required. In this improved technology, the extraction thimble is initially lowered directly into the flask containing the boiling solvent to remove residual extractable material while the extractable materials pass readily from the sample and dissolve into the solvent simultaneously. The level of solvent is then reduced to a level below the extraction thimble such that the configuration mimics the traditional Soxhlet extractor whereby the PAH is extracted by refluxing condensed solvent and collected in the solvent below the extraction thimble. With this improvisation, the PAH extraction efficiencies and precisions were statistically improved, with almost 100% recovery rates [14]. In addition to that, the compact design of the automated system also allows several samples to be extracted simultaneously with its multiple extraction cells assembly while being run unattended [4, 5].

### 2.2. Ultrasonic Agitation/Sonication

The ultrasonic agitation, also known as sonication, is a technique which engages the acoustic energy of ultrasonic waves with a minimum frequency of 16 kHz in fluid, causing rapid compression and rarefaction of fluid movement which results in the cavitation phenomenon, that is, the reoccurring formation and collapse of microbubbles. This agitation can be performed either by immersing a sonicator transducer also known as an ultrasonic horn into the sample solvent mixture or placing the sample solvent mixture directly into a sonication bath. The desired ultrasound is generated by means of piezoelectric ceramic attached either to the ultrasonic horn or the walls of the sonication bath.

Sun et al. [15] claimed that sonication was better than the Soxhlet because it provided higher extraction efficiencies, was more economical and easily operated. Likewise, Guerin [4] noted that similar levels of extraction efficiency to the Soxhlet extraction method could be attained through vigorous sonication. However, the level of extraction efficiency was observed to be highly dependent on the sample matrix and concentration of contaminants in the sample. Contrary to these observations, other studies have indicated that sonication was less efficient than the Soxhlet with relatively low recoveries particularly for lower molecular weight PAHs (44–76%) [13, 16].

 The power amplitude and duration of sonication need to be carefully controlled in order to avoid extensive exposure to the irradiation which may degrade the contaminants in the sample and reduce the extraction rates of PAHs. The decrease in efficiency during excessive sonication is due to an increase in broken carbonaceous particles and additional contact surface area which adsorbs the PAHs more readily, causing a reversed adsorption cycle of PAHs [16]. Additionally, further separation techniques such as centrifugation or filtration are required after the extraction process.

### 2.3. Mechanical Agitation

This simple, low-cost method uses agitation or mixing action to extract the PAHs from samples in a shake-flask placed onto a rotary shaker, or with a magnetic stirrer submersed into the flask directly. Although it is an easy handling method with minimal glassware and smaller volumes of extraction solvent, this method has not been as widely used as the Soxhlet and sonication due to the lower extraction efficiency and unsatisfactory quantitative results [5, 7]. Although some studies reported that this method was comparable to the Soxhlet technique, the results obtained using mechanical shaking showed larger variations and less selectivity due to the difficulty in quantifying the PAH extracts [12, 17]. Comparable results were only attainable with long shaking times to extend the contact time with solvent [18, 19].

### 2.4. Accelerated Solvent Extraction (ASE)/Pressurised Fluid Extraction (PFE)

Accelerated solvent extraction (ASE) or pressurised fluid extraction (PFE) is a fairly new technology which raises the solvent temperature above its boiling point but maintains it in the liquid phase by elevating the pressure. As a result, the high pressure aids in the solubilisation of air bubbles, thereby exposing more of the sample to the extraction solvent while increasing the capacity of the heated solvent to impart better solubility.

Today, ASE systems are commercially available for extracting organic compounds from a variety of solid samples. The ASE system is built up of several extraction cells on a loading tray proximate to an oven. During extraction, organic solvent is pumped into the extraction cells preloaded with soil samples while increasing the temperature and pressure to the desired values. Once extraction is completed, a nitrogen cylinder is used to purge the samples of residual solvent.

 With the usage of the ASE system, the recovery of PAHs from soils and sediments was reported to be two times higher than using the Soxhlet extraction method [20], while the accuracy was also improved with a relative standard deviation of less than 10% [21]. Other benefits of ASE include reduction of solvent consumption and total time required due to the use of high pressures. The extraction procedure can be fully automated with an online purification column, preventing loss of the volatile PAHs, avoiding tedious preparation and potential contamination as in the case of mechanical shaking [21, 22].

### 2.5. Supercritical and Subcritical Fluid Extraction

Supercritical fluids exhibit a continuum of both gaseous and liquid phase properties. Their physical characteristics including liquid-like density, low viscosity, high diffusivity and zero surface tension enable them to penetrate almost anything and dissolve most materials into their components. Carbon dioxide which has a supercritical temperature and pressure of 31°C and 74 bar, respectively, is widely employed in SFE as an environmentaly friendly solvent in its supercritical state [23].

In a study by Miége et al. [9], comparisons between Soxhlet and SFE extraction revealed that the recoveries of PAHs for both methods were almost similar. Although the SFE technique was more difficult to optimise, the technique provided extraction results with lower relative standard deviation and better selectivity, due to cleaner extracts. Other studies [6, 23] also indicated that SFE removed only 8% of the bulk organic matrix in comparison with Soxhlet extraction or ASE which extracted a quarter to one third of bulk soil organic matter. Furthermore, integrated SFE systems allow concentrated extracts to be directed straightaway into the cleanup column, reducing the need to remove the eluate manually. In certain SFE systems, the extracts may also be analysed directly by GC without any cleanup. This prevents extra contamination that may occur during manual handling [12, 24]. However, the high complexity of the SFE process may contribute to inconsistent results this system should be carried out in different laboratories [23].

 In the development of SFE, water has also been considered as the extraction fluid. However, the use of supercritical water is limited because of the high temperature (>374°C) and pressure (>218 atm) requirements which creates a highly corrosive environment [25]. Thus, subcritical water extraction (SWE) also known as pressurised hot-water extraction is used instead. As the temperature of water is raised from 100°C to 274°C under pressure, the hydrogen bonding network of water molecules weakens resulting in a lower dielectric constant and simultaneously decreasing of its polarity. Thus, subcritical water becomes more hydrophobic and organic-like than ambient water, promoting miscibility of light hydrocarbons with water [26]. In contrast to SFE which extracts mostly non polar organic compounds, it has been reported that SWE gives better preference to more polar analytes, therefore providing a higher extraction efficiency of PAHs with less or almost no extraction of other alkanes [6]. Wet oxidation or SWE combined with oxidation using oxidising agents such as air, oxygen, or hydrogen peroxide was reported to remobilise bound organic residues, providing a higher extraction capability [27, 28]. In one study, SWE combined with oxidation resulted PAH soil extraction efficiencies within the range of 99.1–99.99% compared to extraction efficiencies within 79–99+% using SWE alone [28].

### 2.6. Microwave-Assisted Extraction (MAE)

Another highly instrumental extraction technique is the MAE whereby both solvent and samples are subjected to heat radiation energy attained from electromagnetic wavelengths between 1 m and 1 mm, with frequencies of 300 MHz to 300 GHz. Microwave radiation is preferred compared to conventional heating due to its rapid heating which is reproducible and has less energy losses. Modern designs of the microwave ovens include carousels which can hold at least twelve extraction vessels allowing simultaneous multiple extractions. The main advantages of the MAE method are the reductions in solvent usage and time. In comparison to SFE, the cost of MAE is moderately lower [20]. Additionally, this unique heating mechanism provides selective interaction with polar molecules which greatly enhances the extraction efficiency of PAHs [29, 30]. 

 The major drawback of this method however is that the solvent needs to be physically removed from the sample matrix upon completion of the extraction prior to further analysis. In certain cases whereby samples are pretreated with activated copper bars to assist the extraction process, the removal of this copper is necessary for a cleaner extract [10]. Although a subsequent purification step can be implemented to rectify this problem, there may be possibilities of losing analytes or inducing contaminants with additional cooling time for this extra handling. Furthermore, the sample allowance for analysis is limited to 1.0 g which is insufficient for a homogenous analysis [31].

### 2.7. Alternative PAH Extraction Techniques

Solid phase extraction (SPE), a method that is generally used to clean up a sample has been used for rapid and selective extraction of PAHs from soil samples. Soil samples are washed with solvent to leach away undesired components before extraction of PAHs with a different solvent into a collection tube [32]. When this extraction technique is employed, filtering over an empty SPE column or using purified sand prior to extraction is usually recommended to prevent soil samples clogging the SPE column. A variation to the SPE of PAHs from soils is the solid phase microextraction (SPME). Ouyang and Pawliszyn [33] described the application of the technique on PAH extraction from soils. This solvent free approach utilises a small diameter fused-silica fibre coated with the extracting phase and mounted in a syringe-like device for protection and ease of handling. The depth of injected needle is adjusted for headspace sampling before exposing the fibre which adsorbs the PAHs from the soil. The exposed SPME fibre is then transferred directly to the injection port of an analytical instrument such as a GC for quantitative analysis. The major advantage of the SPME is its fast, simple and convenient extraction which can be done on-site. The configuration of the solid-phase microextractor offers solutions to sampling problems because it allows extraction of small volume of samples which can then be analysed without any pretreatment. When stored properly, the fibre on the needle can also be analysed several days later in the laboratory without significant loss of volatiles. The capability of the SPME device to extract such small volumes of samples requires extreme precision during manufacturing to achieve homogeneity in the construction of the fibre (extraction phase surface) to provide consistency in extraction outcomes and qualities [34]. One study using SPME revealed that only volatile compounds such as lower molecular weight (LMW) PAHs (less than 4 rings) were detected [35].

Another alternative PAH extraction technique is thermal desorption which does not use solvents or high-pressure extraction equipments. The thermal desorption technique is commonly coupled with GC by direct injection of solid sample onto the cold injector. The carrier gas is temporarily halted while the injector is rapidly heated to the desired temperature approximately within 200–500°C to volatilise targeted compounds from soil. The carrier gas is then resumed and the isothermally extracted compounds are swept onto the GC column, providing a direct and rapid analysis of the contaminated soil. Thermal desorption and online GC analysis technique has been widely employed in the analysis of PAHs in various matters including fly ash, ambient air particulate matter as well as creosote and petroleum contaminated soil [36–39]. The technique, however, requires prior calibration to allow for nonlinear response to sample size and concentration of contaminants [39].

 Contrary to thermal desorption, pyrolysis (Py) or high temperature distillation (HTD) extraction technique employs high rate temperature ramping or flash pyrolysis at high temperatures. In flash pyrolysis, the sample is heated in a very short time using either inductive heating (also known as Curie point pyrolysis) or Ohmic heating using platinum foil. The significant increase in heat energy in the system causes thermal cracking of larger macromolecules into simpler monomers which are more volatile. Due to its high heating velocity, accurate temperature reproducibility and wide temperature range, the Py has successfully been applied to various nonvolatile compounds and matrices such as synthetic plastics, rubbers and paints. Buco et al. [40] have demonstrated its novel application in the analysis of PAHs in contaminated soil. Here, induction heating of the soil sample is carried out in a ferromagnetic foil called pyrofoil in an oven equipped with a radio frequency field to reach the Curie point temperature (160–1040°C) whereby the pyrofoil loses its magnetic properties and simultaneously adopts the specific property of a heated alloy. As such, the soil sample which is wrapped inside the pyrofoil is desorbed of the PAHs and the PAH bearing pyrolysates are transferred immediately into an online GC column for further analysis. Pyrolysis methods have been a more popular choice than thermal desorption due to their capabilities in providing greater temperature control. With high temperature Py method, the extraction speed is also significantly reduced, permitting a higher number of samples to be analysed. The main advantages of thermal desorption or pyrolysis with online GC is the exclusion of reconcentration and clean-up steps necessary for some other extraction methods. Therefore, the contamination risks are lower with higher sensitivity and specificity when these methods are employed. Similar to SPME, the use of solvents are also eliminated, which subsequently reduces cost. Nonetheless, the small sample size used (approximately 30 mg) may result in insignificant data analysis errors since it does not provide a good representative of the entire field soil. In addition, the temperature program used has to be carefully optimised to avoid the decomposition of the cellulose filter itself, which may result in formations of undesirable byproducts.

 Fluidised-bed extraction (FBE) has also been reported in the specialised literature to extract PAHs from soils [41]. The system is analogous to the automated Soxhlet extraction apparatus whereby the soil sample is loaded into an extraction tube secured with a filter at the bottom while the extraction solvent is filled into the basic vessel beneath the soil sample. The heating block of the device is first heated up to evaporate the extraction solvent through the filter which then condenses when in contact with the cooling bar above the soil sample. The condensed solvent then drips back into the soil sample and further down into the collected solvent. The constant penetrating flow of solvent vapour heats up and agitates the soil mixture, causing it to be fluidised. The collected solvent in the basic vessel is then concentrated for further analysis. In comparison with the conventional Soxhlet extraction, the extraction duration and solvent used is reduced under optimised conditions.

## 3. Influencing Factors

### 3.1. Temperature

In the majority of analytical studies using ASE, SFE, and MAE, the PAH extraction efficiencies were observed to generally increase with increasing temperatures, as can be seen in Table 1 [9, 31, 42–46]. Elevated temperatures reduce both fluid density and viscosity, resulting in lower surface tension and improved contact between the solvent and targeted PAH analytes. The diffusion of PAHs through the soil as well as the diffusion of solvent into the interior of the soil matrix is enhanced. Likewise, the desorption of PAHs from the solid matrix and their solubilities in the extraction solvent are improved by increased temperatures. As such, the time to achieve equilibrium is significantly shortened. Unfortunately, 2- and 3rings PAHs are highly volatile and more susceptible to evaporation instead of extraction at higher temperatures. Thus, the reported extraction efficiencies for LMW PAHs were less than the higher molecular weight (HMW) PAHs. A few papers reported that increasing temperatures caused a general decrease in the PAH extraction efficiencies and recovery yields [44, 47]. While there is no certain explanation for this behaviour, it has to be noted that these studies were using SFE.

### 3.2. Solvent Type

Table 2 is a bibliographic compilation of PAH extraction studies from soils using various solvents. Generally, the choice of extraction solvent is dependent on several factors, with one of them being the degree of PAH concentration in the soil. For lowly polluted soil (≈ *μ*g/kg dry weight sample), PAHs are mainly found on the surface, therefore a more polar solvent such as acetone is preferred to break up the soil aggregates and to allow intensive contact between particles. For highly polluted soil (≈ mg/kg dry weight sample) however, a relatively nonpolar solvent such as toluene or cyclohexane would be a better solvent [12]. Since the principles of solvent extraction are based on the theory of like dissolves like, the polarity of solvent with respect to the polarity of PAH contaminants also plays a role in determining the extent of solubility. For instance, it was shown that dichloromethane as an extraction solvent for PAHs resulted in low recoveries for all compounds, whereas hexane-acetone (1 : 1) was an effective extraction solvent for PAHs [48, 49]. Apart from PAH concentration and polarity of solvents, extraction efficiencies vary from one technique to another. In MAE, for example, solvents are chosen based on their dissipation factor (dielectric constant) which determines the degree of absorption of microwave energy [31, 49].

### 3.3. Soil Moisture and Other Soil Characteristics

The effects of soil moisture on PAH extraction efficiencies are dependent on the type of extraction technique employed as shown in Table 3. With MAE studies, PAH extraction efficiencies were generally observed to increase with increasing soil moisture. This is mainly due to the ability of the localised superheating to form gas bubbles from existing water residues in soil and cause expansion of pores, allowing solvent penetration into the matrix. Additionally, the high dielectric constant of water allows more microwave absorption which in turn provides more heating [29, 30]. Similarly, a study using SFE showed that for water content less than 10 wt. %, the water in soil acted as a modifier to the extraction solvent which increased the fluid's capability to penetrate further into the soil particles [50]. Other SFE and Soxhlet experiments revealed that the presence of soil moisture decreased or did not significantly affect the efficiency of PAH removal from soil. Soil drying is therefore carried out in some cases to eliminate the influence of moisture on the PAH extraction efficiency. Comparisons between various drying methods showed that thermal drying of soil between temperatures of 25°C and 40°C for several days was best for prevention of losses of volatile PAHs while air drying was reasonably sufficient and freeze drying was least preferable due to partial loss of highly volatile PAHs such as naphthalene [12].

Apart from soil moisture, the composition of soil affects the extraction of PAHs. The extraction process of PAHs was observed to be significantly more difficult from high clay content soil (>40%) due to the fact that 32% of the total carbon content where most of the HMW PAHs resided in was concentrated in the clay fraction [17]. Strong adsorption of PAHs to clay surfaces also result in reduced desorption during thermal extraction and less detectable hydrocarbons [39]. The size of soil particles also impacts the efficiency of PAH extraction. It has been demonstrated that PAHs accumulate preferentially on smaller particles [41]. As such, PAHs are more easily extracted from fine soil fractions such as fine silts and clays than larger aggregate size fractions. Reduced particle sizes allow ample diffusion and better accessibility of solvent through the matrix, thus increasing the flow rate of solvent and rate of extraction [17, 51].

## 4. Kinetics Models of PAH Desorption from Soils

### 4.1. First-order Mass Transfer with Single Equilibrium Desorption

The dissolution and desorption of PAHs can be fitted to a first-order mass transfer coefficient model [52]:

(1) $$C_{w}=C_{e}\left[1-\exp\;\left(-kt\right)\right],$$

where *C*_*w*_ is the liquid-phase concentration at any point in time, *k* is the lumped mass transfer coefficient, *C*_*e*_ is the equilibrium liquid-phase concentration and *t* is the contact time with the extraction solvent.

### 4.2. First-order Mass Transfer with Dual Equilibrium Desorption

The desorption process in sediments and soils contaminated with hydrophobic contaminants can be classified as a biphasic process, with a fast and a slow component [53–55]. This two-site kinetic model is described by

(2) $$C_{w}=C_{e}-C_{1}\exp\;\left(-k_{1}t\right)-C_{2}\exp\;\left(-k_{2}t\right),$$

where *C*_*w*_ is the liquid-phase concentration at any point in time, *C*_*e*_ is the equilibrium liquid-phase concentration, *C*_1_ is the equilibrium liquid-phase concentration of the first stage (rapid), *k*_1_ is the mass transfer coefficient of first stage, *C*_2_ is the equilibrium liquid-phase concentration of second stage (slow), *k*_2_ is the mass transfer coefficient of second stage, and *t* is the contact time with the extraction solvent.

This model treats the process as a combination of two kinetically controlled reactions occurring simultaneously, whereby the first stage is governed by a rapid partitioning between the solid and liquid phases while the latter stage is which generally slower than the first is kinetically controlled by other processes. Equation (2) can also be employed in its fractional form whereby the rapidly desorbing fraction is *φ*_*s*_ while the slowly desorbing fraction is (1 − *φ*_*s*_):

(3) $$\frac{C_{t}}{C_{o}}=1-\left[\varphi_{s}\exp\;\left(-k_{1}t\right)\right]-\left[\left(1-\varphi_{s}\right)\exp\;\left(-k_{2}t\right)\right],$$

where *C*_*t*_/*C*_*o*_ is the fraction of the PAH extracted after time *t*.

## 5. Conclusions

Of the PAH extraction technologies discussed here, Soxhlet extraction, ultrasonic and mechanical agitation can be implemented easily since the processes are carried out with minimal instruments or glassware and at ambient pressures. In comparison, ASE and MAE provide a faster extraction with lesser solvent consumption albeit at higher capital costs and possibly operating costs. PAH extraction using supercritical carbon dioxide or subcritical water is an environmentaly friendly technique but entails the use of high pressure equipment. SPE and SPME, thermal desorption and flash pyrolysis, as well as fluidised-bed extraction are novel alternatives which require further in-depth studies prior to wide-scale adoption in laboratories. It has to be recognised that no single extraction technology can be the solution for all extractions of PAHs in soils and sediments. Costs, the required accuracy and precision in results, analysis time, as well as technical competence are factors to be considered in deciding the right extraction technique.

## Figures and Tables

**Table 1:** Bibliographic compilation of studies on extraction temperature.

Extraction technique	Temperature (°C)	PAHs studied	Effect of increasing temperature on PAH extraction efficiency (+/−)^(a)^	Reference
SFE	80, 100, 120	16 PAHs	+	[9]
MAE	70, 100	16 PAHs	+	[31]
ASE	70, 90, 175, 200	Naphthalene, pyrene	+	[42]
ASE	20, 40, 60, 100, 150	Acenaphthene, pyrene	+	[43]
SFE	80, 100, 120	Phenanthrene	+ in some case; − in others	[44]
MAE	80, 115, 145	17 PAHs	+	[45]
MAE	35, 50, 65, 80, 95	Fluorene, phenanthrene, anthracene, fluoranthene, pyrene	+	[46]
SFE	50, 80	Naphthalene, anthracene, pyrene, chrysene, benzo[a] pyrene, indeno[1,2,3-c,d] pyrene	−	[47]

^(a)^+: PAH extraction efficiency increased; −: PAH extraction efficiency decreased.

**Table 2:** Bibliographic compilation of solvents used in the extraction of PAHs.

Extraction technique	Solvent	PAHs studied	Solvent(s) with high PAH extraction efficiency	Reference
Sonication	Acetone, cyclohexane, 2-propanol, methanol, dichloromethane, acetonitrile	16 PAHs	40% acetone in water	[15]
FBE	Cyclohexane-acetone (90 : 10 and 30 : 70), n-hexane-acetone (90 : 10 and 40 : 60), cyclohexane, n-hexane	16 PAHs	Cyclohexane, n-hexane	[41]
MAE	Hexane, dichloromethane, acetonitrile, acetone, hexane-acetone (1 : 1)	16 PAHs	Hexane-acetone (1 : 1)	[49]
SFE	Cyclohexane-acetone (1 : 1), hexane-acetone (1 : 1), dichloromethane	Naphthalene	Hexane-acetone (1 : 1)	[50]

**Table 3:** Bibliographic compilation of studies on soil moisture.

Extraction technique	Soil moisture (wt. %)	PAHs studied	Effect of increasing moisture on PAH extraction efficiency (+/−/n.d.)^(a)^	Reference
MAE	Dry, 30	24 PAHs	LMW PAHs: n.d.; HMW PAHs: +	[29]
SFE	0–40	Phenanthrene	−	[44]
MAE	Dry, 20	16 PAHs	n.d.	[45]
MAE	Dry, 18.5	16 PAHs	+	[49]
Soxhlet			n.d.	
SFE	<10	Naphthalene	+	[50]
	10–20		−	

^(a)^+: PAH extraction efficiency increased; −: PAH extraction efficiency decreased; n.d.: no significant difference.
